# Renal profile and the associated outcome of patients with acute kidney injury undergoing dialysis in Renal Unit at a Tertiary Healthcare Facility in Western Kenya

**DOI:** 10.4314/ahs.v25i1.30

**Published:** 2025-03

**Authors:** Rodgers Norman Demba, Sylviah Mweyeli Aradi

**Affiliations:** 1 Department of Medical Laboratory Science, School of Medicine, Maseno University; 2 Department of Internal Medicine, School of Medicine, Maseno University

**Keywords:** Renal Profile, Acute Kidney Injury, Dialysis and mortality

## Abstract

**Background:**

Acute Kidney Injury (AKI) is characterized by sudden decline or loss of kidney function.

**Objective:**

This study aimed to determine the renal profile and the outcome of AKI patients undergoing dialysis.

**Methods:**

Retrospective cohort study was conducted from January 2015 to December 2021.

**Results:**

Of the 69 AKI patients enrolled in this study, 33 (47.8%) were men, and 36 (52.2%) were women. Majority (55.56%; 10) of the AKI patients died within one month of diagnosis, 44.44% (8) died after one month. Independent predictors of survival were creatinine level (adjusted hazard ratio= 20.54, 95% CI: 0.15, 2862.17; P = 0.23), urea level (adjusted hazard ratio= 0.56, 95% CI: 0.05, 6.78; P = 0.65), phosphate level (adjusted hazard ratio= 5.94, 95% CI: 0.51, 69.57; P = 0.16), calcium level (adjusted hazard ratio= 0.31, 95% CI: 0.04, 2.67; P = 0.29), sodium level (adjusted hazard ratio= 0.53, 95% CI: 0.27, 10.21; P = 0.67), potassium level (adjusted hazard ratio= 5.3, 95% CI: 0.38, 73.56; P = 0.21), chloride level (adjusted hazard ratio= 0.23, 95% CI: 0.03, 1.66; P = 0.15).

**Conclusion:**

AKI patients had a mortality rate of 26% after initiation of dialysis.

## Introduction

Acute kidney injury (AKI) is a clinical syndrome characterized by sudden decline in kidney excretory function leading to accumulation of end products of urea and creatinine or the loss of urine output^1^-^4^. The incidence of acute kidney injury has been on the rise making it a major public health issue globally, associated with poor outcomes, high mortality rate, severe in-hospital complications, and rise in treatment cost^5^, ^6^. Worldwide, the burden of acute kidney injury with regards to mortality has exceeded by far that of diabetes, cancer and heart failure in the last fifty years from the year 2013^7^.

The mean age of patients with acute kidney injury is sixty years globally, however, in low and middle income countries, there has been a decline to a mean age of fifty years attributed to decreasing socioeconomic status especially among men^8^. Limited access to healthcare or sex related risks has contributed to an increases cases of acute kidney injury among males compared to females^9^. The predominance of Acute kidney injury in high-income countries has been hospital-acquired, whereas, in low-income settings these cases are community-acquired^7^, ^10^. Among different countries, there is a significant difference in the mortality, prevalence and risk factors of acute kidney injury due to varying hospital facilities and different socio-economic statuses^11^. In developing countries, increasing cases of acute kidney injury is attributed to no-adherence to medication^12^, high level of poverty rate^13^, and elevated anxiety levels^14^. Estimating the prevalence and outcome of AKI in many developing countries has been difficult due to restricted resources to conduct research and underdiagnoses^15^. A single-Centre study from Sudan reported that there is no reliable statistics on AKI in Africa, however, the high burden are linked to dehydration, and complications during pregnancy^16^. In Africa the causative agent of acute kidney injury is associated with infections such as malaria and HIV/AIDS, obstetric and surgical complications, nephron-toxins and this results to high cost of management, prolonged hospital stay and inhospital mortality^17^. The risk factors linked to acute kidney injury include socio-economic factors, environmental, patient factors related to care and acute exposure to nephrotoxic drugs, insufficient control of infectious diseases and inadequate healthcare systems^18^.

For diagnosis and screening of acute kidney injury, the best overall index of kidney function is to perform glomerular filtration rate (GFR), however, direct measurement has its challenges, and so GFR is estimated by use of serum levels of endogenous filtration markers such as creatinine and a small increase of serum creatinine is associated with poor prognosis of AKI^19^. In addition, biomarkers of kidney injury such as neutrophil gelatinase-associated lipocalin (NGAL), kidney injury molecule (KIM-1) and urine output test can be used as a sensitive parameter of kidney function 20. In acute kidney injury, serum creatinine and urea nitrogen levels are often used as biomarkers of disturbance in the main function of kidneys which might results to potassium excretion, hyperkalemia, hyponatremia and hypernatremia, impaired phosphate clearance leading to hyperphosphatemia^21^. Collecting urine and blood sample to assess the creatinine clearance can be used as a rapid kidney function test in the case of oliguria^22^,^23^. For early diagnosis of an impairment of kidney function, creatinine clearance test is more precise than an elevated levels of plasma creatinine^23^. Monitoring of urinary biochemistry and urine microscopy can be used to predict the likelihood of an early stage of recovery in patients with acute kidney injury recovery whereas, a combined test of urinary biochemistry of fractional excretion of urea nitrogen (FeU), urinary microscopy and fractional excretion of sodium (FeNa) can facilitate differential diagnosis of AKI^24^. Unpublished data from registry of births and death in western Kenya recorded morbidities and mortalities attributed to acute kidney injury. It is on the premise of this report from registry of births and death in western Kenya that prompted this current study to establish the survival pattern & predictors of mortality in patients with acute kidney injury undergoing dialysis in renal unit at a tertiary healthcare facility in western Kenya.

## Materials and Methods

### Study design and population

This was a retrospective cohort study conducted from January 2015 to December 2021 December 2021. This study analysed data on 69 patients who were diagnosed with acute kidney injury and enrolled for haemodialysis at the renal unit in General Teaching and Referral Hospital, which is a tertiary healthcare facility in Western Kenya. The 69 patients were enrolled between January 2015 and December 2021. Dialysis was first initiated in this tertiary healthcare facility located in Western Kenya in January 2015.

### Data collection

Clinical records of acute kidney injury patients from the renal unit were reviewed, and a consecutive sampling method was used to collect data on AKI patients enrolled at the facility within the study period. Only information necessary to address the objectives of this study were extracted from the registry records. We excluded incomplete data and data on AKI patients who died due to COVID-19 as this was considered a confounder. We considered treatment status and existence of comorbidities as potential prognostic indicators in AKI patients undergoing haemodialysis. Data on treatment and comorbidity status were obtained from the registry records. The levels of creatinine, urea, phosphate, calcium, sodium, haemoglobin, potassium, and chloride were considered as potential predictors of survival among AKI patients. Haemoglobin analysis was carried out by the health facility using the Medonic Hematology Analyzer M32 - Hematology Analyzer (Boule Diagnostics AB, Domnarvsgatan 4 Spanga, and Stockholm, Sweden-based Company). The COBAS INTEGRA 400 plus (Roche Diagnostics, United States) was used for analysis of creatinine, urea, phosphate, calcium, sodium, potassium, and chloride levels. Data were collected from medical records using a paper-based data collection tool (questionnaire) to gather additional information on the 69 patients with acute kidney injury. The additional information collected included the age, gender, survival (deceased or alive and undergoing dialysis), comorbidity, and treatment status of the patients.

### Statistical analysis

Data were analysed using statistical package for social sciences (SPSS) version 23 (SPSS Inc., Chicago, Illinois, United States). Cronbach's alpha coefficient was used to determine the reliability of the data collection tool. For the 12 questions in the questionnaire, the Cronbach's alpha was 0.736, and based on standardized items, the Cronbach's alpha was 0.739. Generally, a Cronbach's alpha value above 0.70 indicates sufficient consistency, suggesting that the measure is reliable. To determine the demographic characteristic influencing the survival of acute kidney injury patients undergoing haemodialysis, descriptive statistics and binary logistic regression analysis were used. To establish haematological and renal biochemical parameters as predictors of survival among patients undergoing haemodialysis, a univariate and Multivariate Cox regression analyses was used to identify independent predictors of time to death. For all statistical analyses, P values less than 0.05 were considered statistically significant. The presence of multi-collinearity was checked using variance inflation factor. The proportionality of hazard assumption was checked using Log (-Log) S (t) plots.

## Ethical considerations

This study obtained ethical approval from Eastern Africa, and National Commission for Science and Technology. Only secondary laboratory data were analysed for this study and we did not require patient identifiers in the data. Patients consent was not required since identifier linked to a patient was redacted by the tertiary facility in western Kenya to maintain confidentiality. In this current study, data was obtained from acute kidney injury patients who were aged 18 years and above. Living patients still enrolled in the study at the time of the study had their identifier censored by the tertiary facility in western Kenya to maintain confidentiality.

## Results

Thirty three (47.8%) of the patients were men, and 36 (52.2%) were women (Table 1). Out of the 69 AKI patients that were undergoing dialysis, 51 (73.9%) were alive and 18 (26.1%) deceased. Among the male patients with AKI, 6 were deceased, while among the women, 12 were deceased. A binary logistic regression analysis indicated that there was no statistically significant relationship between gender and survival status of a patient with AKI (Relative risk = 0.44, 95% CI: 0.14, 1.37; P = 0.15). A P value 0.15 implies that among the AKI patients enrolled in this study, there was no difference in survival between male and female. Out of the 18 deceased patients with AKI, nine (11.1%) were aged 18- 33 years and nine (11.1%) were above 33 years of age. In this current study, there was no statistically significant relationship between age and survival status of a patient with AKI (Relative risk = 1.13 95% CI: 038, 3.3; P = 0.83). A P value of 0.83 denotes that among the AKI patients enrolled in this study, there was no difference in survival between 18-33 years and 33 years and above.

**Table 1:** Cox regression analysis on demographic and clinical characteristics of AKI patients enrolled at a tertiary healthcare facility in western Kenya from January 2015 to December 2021

			Unadjusted (crude) hazard ratio		Adjusted hazard ratio	
Patient characteristics	Survival status (n)					
						
	Alive	Deceased	RR	P value	RR	P value
Gender			2.66 (95% CI: 0.98, 7.2)	0.06	3.42 (95% CI: 0.96, 12.19)	0.58
Male (n=33)	27	6				
Female (n=36)	24	12				
Age groups			1.26 (95% CI: 0.5, 3.18)	0.63	1.16 (95% CI: 0.39, 3.39)	0.78
18 – 33 years (n=36)	27	9				
> 33 years (n=33)	24	9				
Laboratory results Elevated creatinine * (> 106 µmol/L in males & > 26 µmol/L in females) [n = 27]	35	18	28.5 (95% CI: 0.17, 4778.5)	0.2	20.54 (95% CI: 0.15, 28.9)	0.23
Elevated urea * (> 7.1 mmol/L in adults) [n= 58]	41	17	2.62 (95% CI: 0.35, 19.77)	0.35	0.56 (95% CI: 0.05, 6.8)	0.65
Elevated phosphate ** (>1.45 mmol/L in adults) [n= 53]	36	17	4.74 (95% CI: 0.63, 35.62)	0.13	5.94 (95% CI: 0.51, 69.57)	0.16
Elevated calcium **	36	15	1.93 (95% CI: 0.56, 6.67)	0.3	0.31 (95% CI: 0.04, 2.66)	0.29
(>2.5 mmol/L in adults) [n= 51] Low Hb** (< 13.5 g/dL in males & < 12.5 g/dL in females) [n = 64]	46	18	23.1 (95% CI: 0.02, 34652.8)	0.4	8.5 (95% CI: 0.12, 6115.0)	0.52
Elevated sodium * (>145 mmol/L in adults) [n= 54]	37	17	4.13 (95% CI: 0.55, 31.07)	1.68	0.53 (95% CI: 0.03, 10.2)	0.67
Elevated potassium * (>5.5 mmol/L in adults) [n= 40]	25	15	3.71 (95% CI: 1.08, 12.83)	0.04	5.3 (95% CI: 0.38, 73.56)	0.21
Elevated chloride * (>106 mmol/L in adults) [n= 49]	35	14	1.68 (95% CI: 0.55, 5.13)	0.36	0.23 (95% CI: 0.03, 1.67	0.15
Comorbid status [n=68]	51	17	7.73 (95% CI: 1.0, 59.87)	0.05		
Type of comorbidity [n=68]	51	17	1.20 (95% CI 0.79, 1.83)	0.38	1.27 (95% CI: 0.78, 2.07)	0.33
Hypertension [n=14]	12	2	-			
Hypertension & Diabetes mellitus [n=17]	13	4	-			
Treatment status Not on any treatment n= [39]	25	14	2.69 (95% CI: 0.88, 8.16)	0.08	1.58 (95% CI: 0.33, 7.51)	0.57

RR is relative risk, Hb is haemoglobin

* Testing done every two weeks

** Testing done monthly, P value < 0.05 was considered statistically significant

To monitor the kidney function overtime with a view of establishing if the condition of the kidney is getting worse or better, a panel of laboratory test was done to AKI patients prior to dialysis. The unadjusted (crude) univariate Cox regression analysis showed that treatment status, type of comorbidity, hypertension, elevated levels of calcium, phosphate, sodium, chloride and low level of haemoglobin were not statistically significant indicators in determining the survival of AKI patient except for potassium (table 1). Furthermore, the relative risk of dying if one had elevated levels of creatinine, phosphate, potassium, and low level of haemoglobin was above^1^.

However, the adjusted Multivariate Cox regression analysis showed that none of the renal functions test, haemoglobin level, and comorbidity status were statistically significant indicators in determining the survival of AKI patient suggesting that AKI patients at a tertiary healthcare facility in Kenya. Sixty eight of the enrolled acute kidney injury patients had comorbidities for which they were also receiving clinical care. The recorded chronic diseases included hypertension^14^ (20.59%), hypertension and diabetes mellitus 3 (4.41%), pre-eclampsia 11 (16.18%) other comorbid including but not limited to HIV 40 (58.82%) and 1 patients were recorded not to have any comorbid (table 1). Comorbidity status did not significantly indicate a poor survival outcome, however, the P value 0.05 is suggestive a like hood that comorbid might have an influence of the survival status of the AKI patients as shown in table 1. Acute kidney injury patients on treatment, whether to control for comorbid or on iron or calcium supplement had survival outcomes that were statistically significant. On the other hand, the AKI patients who were not on the aforementioned treatment were 2.69 more likely to have poor survival outcomes. At the end of this study period, 51 AKI patients were alive and undergoing dialysis, whereas 18 were reported to have died. Out of the 18 deceased patients who had AKI, 55.6%^10^ died in less than one month following initiation of dialysis, 44.6%^8^ died after one month.

## Discussion

Our findings demonstrated that age, gender, renal biochemical parameters used to monitor kidney function, haemoglobin level, treatment status and comorbidity status were not statistically significant indicators in determining the survival of acute kidney injury patient. However, the relative risk association exhibited that acute kidney injury patients with deranged creatinine, phosphate, potassium, presences of comorbidity, haemoglobin, and poorly controlled comorbid were more likely to have poor survival outcome. This implies that early recognition of renal impairment, monitoring renal function through laboratory investigations, initiation of dialysis and intensive medication use may be useful in the recover to normal or nearly normal kidney function. During the six year study, 18 out of 69 acute kidney injury patients died, equating to a 26%% mortality rate. The overall mortality rate in our study was consistent with the overall mortality rate of 26.9% among nonsurgical patients and this significantly increased to 35.7% in patients who developed acute kidney injury during their medical intensive care unit from January to June 2021 in Jordan University Hospital^25^. It is worth noting that the mortality rate of this present study are considerably less than the mortality rate of 50-70% in acute kidney injury patients who had severe trauma and were admitted to intensive care unit between August 2002 and September 2007 at a University hospital in de Santo António in the city of Porto in northern Portugal^26^. In another study, the report on mortality rate was at 19.7% and this was from a prospective observational study on the outcome of acute kidney injury patients in Critical Care Unit, from February 2010 to August 2010 in a tertiary referral center in Iran^27^. In other studies, the acute kidney injury was associated with a mortality rate of 20.4%^28^, mortality rates of 36.9% was reported in Cameroon^29^, 44.4% in Malawi^30^, 34.1% in Rwanda^31^ and 58% in 35% in the Democratic Republic of Congo^32^. The overall in-hospital mortality rate was 12.8% following a study on Mortality and predictors of acute kidney injury in adults which was a hospital-based prospective observational study conducted conducted from April to August 2019 at the medical ward of Jimma Medical Center, Southwest Ethiopia^33^. In Sub-Saharan Africa (SSA), the proportion of patients with acute kidney injury is not well known, however, the high mortality are associated with poor access to healthcare^34^-^36^. The variations in the mortality rates of acute kidney injury has be attributed to difference in patients admission sites while at the hospital ranging from intensive care unit, those in general medical ward or in surgical ward to the study design adopted by different authors and also underlying medical conditions of the patients with some having history of chronic kidney disease^33^. This study hypothesizes that patients conditions, and severity of the illness might have contributed to the mortality rate. We also found that risk factors for acute kidney injury include socioeconomic and or cultural factors, environmental, insufficient control of diseases, patient-related factors which can be modifiable for example diabetes mellitus, hypertension, pre-eclampsia, old age, pre-existing chronic kidney disease^18^. In high income countries, acute kidney injury is more frequent in the context of hospital-related risk factors in cases of major surgery, drug toxicity in older patients with multiple diseases, bleeding or septic shock and a milder form of AKI can also be witnessed following community acquired infection^37^-^39^. Contrast in low and middle income countries, acute kidney injury may be caused by HIV infection, malaria or dengue disease, Hantavirus infection^38^,^40^.

## Conclusion

The current study highlights that Acute kidney injury patients had a mortality rate of 26% after initiation of dialysis. Early diagnosis and nephrological care will avert premature deaths in these patients. A well controlled comorbids among acute injury patients undergoing dialysis substantially increases the survival advantage of dialysis.
